# Influence of the Pulse Mode of Manual Metal Arc Welding on Weldment Distortions

**DOI:** 10.3390/ma17205067

**Published:** 2024-10-17

**Authors:** Nikolay Ferdinandov, Danail Gospodinov, Mariana Ilieva, Rossen Radev, Georgi Hristov

**Affiliations:** 1 Department of Materials Science and Technology, University of Ruse “Angel Kanchev”, 8 Studentska St., 7017 Ruse, Bulgaria; nferdinandov@uni-ruse.bg (N.F.); dgospodinov@uni-ruse.bg (D.G.); rradev@uni-ruse.bg (R.R.); 2 Department of Telecommunications, University of Ruse “Angel Kanchev”, 8 Studentska St., 7017 Ruse, Bulgaria; ghristov@uni-ruse.bg

**Keywords:** welding distortions, pulsed welding, manual metal arc welding, 3D scan

## Abstract

As a result of the thermo-mechanical impact during welding, distortions are generated in welded structures. These distortions significantly influence the geometric and dimensional accuracy of welded structures, in many cases lowering their working characteristics and reliability. An optimal design for welded structures is a prerequisite for increased reliability and reduction in manufacturing cost, and such an optimal design can be achieved knowing the distortions in weldments. Despite the fact that pulsed metal inert gas welding and metal active gas welding have been broadly applied in the last few decades, nowadays, few manufacturers, for instance, Fronius, EWM, Redco, and Perfect Power Welders, offer such an option for manual arc welding. This work aims to determine the influence of the parameters of pulsed welding modes on distortions that are generated during manual arc welding. Two different inverter welding power sources were used, and the welding distortions were measured by 3D scanning. The results showed that the pulsed mode during manual arc welding led to a reduction in distortions compared to the conventional welding mode. The crucial part of the manual welding system proved to be the qualification and performance of the welder.

## 1. Introduction

Most of the welding methods use heat, delivered by an external energy source, usually an electric arc. Some of these welding methods are metal inert gas (MIG) welding, metal active gas (MAG) welding, manual metal arc (MMA) welding, tungsten inert gas (TIG) welding, and submerged arc welding (SAW). Other methods, such as friction stir welding, diffusion welding, electric resistance welding, etc., use heat generated inside the welded parts. Both these groups of welding methods are characterized by a nonuniform temperature distribution inside the welded parts because of the restricted volume of the welded metal [1]. Due to the irregular metal heating during welding, thermal expansion and contraction arise at different areas of a weldment, and in this way, welding displacements and distortions are generated. Phase transformations can also contribute to the observed after-welding changes in the dimensions and shape of weldments [2,3]. The scale of welding distortions is defined by the extent and distribution of heat input and the properties of the welded metal [4].

Welding distortions display themselves as shape and dimensions changes, thus alternating the accuracy of structures, making these structures hard to assemble. As residual stresses accompany welding distortions, worsening in the corrosion behavior of weldment is observed too. These are only some of the negative results from welding distortions [2,5]. To overcome the implications of the welding distortions and to increase the structures’ reliability, knowledge and understanding of welding distortions is essential [6,7].

The accuracy of a welded structure depends on the welding performance that is often negatively impacted by insufficient accuracy of blanks, different post-welding operations that disturb the internal forces equilibrium, and most crucially, the peculiarities of the welding process [4].

There are different practical ways to reduce welding distortions [1,2,4,8,9,10] and the most crucial is the correct welding sequence. Some other means to avoid welding distortions include symmetrical placement of weldments, suitable parts’ preparation for smaller bevel angles, appropriate fixation of welded parts by suitable devices, discontinuous welds for thin-walled structures, and the appropriate welding method, combined with appropriate welding parameters, prior deformation of the structural elements in a direction opposite to the expected distortions, and restriction in weld size up to the required for mechanical strength. As welding distortions arise because of nonuniform heat distribution during welding, they can be avoided by welding less, using less heat, and removing the heat [11].

Pulsed arc welding was developed in the 1960s to control material transfer for MIG welding of aluminum [12]. Nowadays, pulsed modes of welding are mainly applied with TIG and MIG/MAG methods [12,13]. Pulsed modes of welding allow a possibility for precise control of the heat input, which is especially important when welding thin plates and high-strength steels, higher process effectiveness, lack of splashes, non-fusion, partial penetration, and weld leaks [12,14,15]. A disadvantage of pulse welding is that the harder the adjustment of welding mode, the greater the increase in the noise load, and significant deviation in the strength of the arc light can be noted.

Pulsed arc welding is mainly applied to the mechanized welding methods and its implementation in manual metal arc welding is a challenge for the manufacturers of welding inverter power sources [16]. Currently, few manufacturers of welding equipment offer power sources working in a pulsed mode. Some of these manufacturers are Fronius [17] with their Trans Pocket 180 power source, Redco [18], which manufactures ASTRA 200 AC/DC PULSE LCD, EWM [19] with Pico 220 cell puls, and Perfect Power Welders [20] with ARC-200DPS Pulse MMA Welder. These manufacturers promote pulsed manual metal welding as a method, suitable for welding in positions different from normal (flat) positions as the welding of corner joints in the PB position allows higher welding speeds to be reached or a lower average current to be used—this is connected to a decrease in heat input, which leads to less distortion. Among the advantages of pulsed MMA is the possibility of the welding of deformed steel pipes or thin plates and materials with a higher weld pool viscosity as it leads to less heat input and greater control of the welding pool, which is outlined. The manufacturers also state that a constant welding speed is easy to maintain as the impulse frequency determines the welding rate, residual sprinkles are reduced, and penetration is full.

Unfortunately, evidence of the advantages of pulsed manual metal welding in the scientific literature is limited. Information on suitable welding parameters and their influence on welding distortions is scarce, such as works [16,21,22]. The authors of [16,21,22] state that the current frequency should not go beyond 5 to 10 Hz, as higher frequencies hinder the control of the welding pool due to the reduction in the temperature difference of the weld pulse during base and peak time.

The aim of this work is to determine the influence of the pulsed modes of operation on distortions during manual arc welding. To accomplish this aim, two different modern inverter power sources were used—TransPocket 180 and ASTRA 200 AC/DC PULSE LCD.

## 2. Materials and Methods

Sheets of S235JR EN 10025-2:2019 steel were used for the experiment. The chemical composition of S235JR EN 10025-2:2019 [23] steel, according to EN 10025-2:2019, is shown in Table 1, together with the chemical composition, determined by spectral analysis.

S235JR steel is an extensive part of welded materials, used in practice, and has the advantage of lacking welding distortions caused by phase transformations. This makes S235JR steel suitable for the experiments presented here as the welding distortions should be a function of thermal stresses only; thus, using S235JR steel as a material for specimens’ production allows us to avoid volumetric changes caused by phase transformations [24,25].

**Figure 1:**
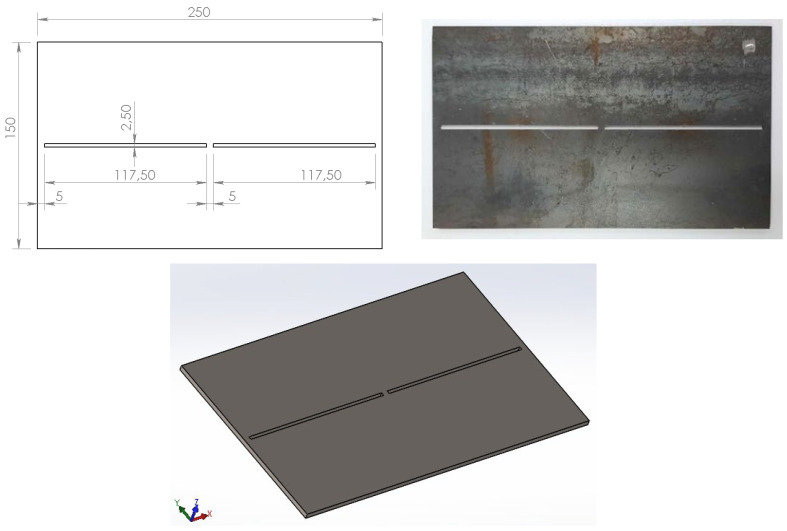
A draft and appearance of the specimens.

The appearance and dimensions (250 × 150 × 4 mm—length × width × thickness) of the specimens are shown in Figure 1. To determine these dimensions, preliminary experiments were carried out to specify a length that allows us to weld a weldment with full penetration by a single pass butt weld, using only one electrode with a length of 350 mm, which is inserted enough to easily measure distortions. These preliminary experiments gave a maximum specimen length of 260 mm. Another result of the prior experiments was the determination of the welding gap length. A continuous welding gap of 2.5 mm that complies with the recommendations of EN ISO 9692-1:2013 [26] allowed full penetration only on the first part of the plate. Due to metal expansion during welding, the gap shrank, and the second part of the weld lacked full penetration. To avoid this, the welding groove was disconnected to form a bridge of 5 mm in length at its center. Thus, the welding gap in our experiments was 2.5 mm wide, 235 mm long, and divided into two halves of 117.5 mm. To imitate tack welds at the beginning and end of welds, the groove was cut by laser cutting with an offset of 5 mm from the edges of the sheet.

To limit excessive penetration that would affect distortions, flat ceramic backing strips with the length of the sheets and without a groove were used. After welding and cooling, the backing strips were removed to measure the welding distortions.

Seventeen welding modes were used Three test bodies were welded for each welding mode, and the results were averaged and presented in Section 3 of this work. As the difference in the recorded distortions that arose during non-pulsed welding with both power sources were small, the results were averaged and labeled as welding mode 1.

Lime type covered OK 48.00 electrodes, 3.2 mm in diameter and manufactured by ESAB, were used. According to the manufacturer, the OK 48.00 electrode is a reliable general-purpose electrode for manual metal arc welding of carbon steels, carbon manganese steels, and fine-grained carbon manganese steels with elevated yield strength. OK 48.00 electrode deposits a tough crack-resistant weld metal.

**Figure 2:**
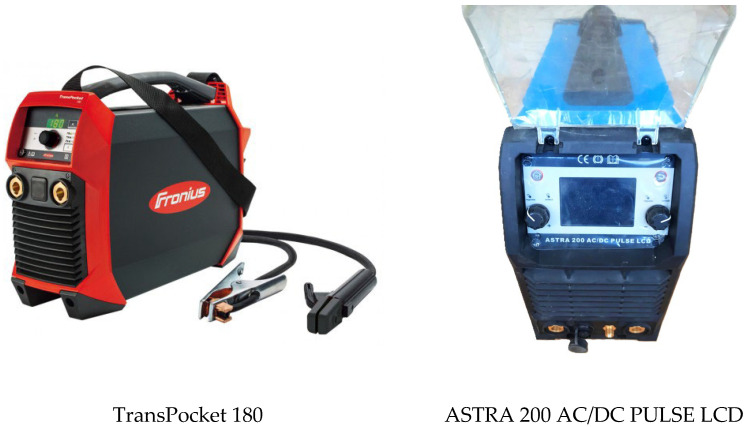
Appearance of the used power sources.

Two different power sources that allow working in pulsed mode were used—TransPocket 180 manufactured by Fronius (referred as “Fronius” in what follows) and ASTRA 200 AC/DC PULSE LCD by Redko (referred as “Astra” in what follows)—see Figure 2 and Table 2. Both can be used for MMA and TIG welding.

The welding current was 120 A, consistent with the diameter of the covered electrode. Welding in direct current reverse polarity mode was used. The frequency of the pulsed current was in the range of 5 to 990 Hz when welding with Fronius, and in the range of 5 to 400 Hz when using Astra. For reference, a welding mode with a constant current was performed too. During welding, the welding time, the arc voltage, and the effective current were measured, using an external voltmeter and ammeter. An experienced welder, qualified according to EN ISO 9606-1 (Qualification testing of welders—Fusion welding—Part 1: Steels) [27]—111 P BW FM1 B t12 PA ss mb, produced all of the specimens. All welds were welded with full penetration.

As Astra offers additional capabilities, the load duty cycle (the ratio between pulse and base current) was altered when welding with this equipment. Table 3 gives all the details of the used welding modes.

Heat input, presented in Table 3, was calculated using the following relation [28]:

(1) $$EN\;Heat\;input=\frac{arc\;voltage\times arc\;current\times termal\;efficiency}{travel\;speed}$$

In MMA welding, thermal efficiency equals 0.8 and the heat input has a dimension of J/mm or kJ/mm [29].

According to [15,30], the relation (1) does not give a correct representation of the heat input in controlled waveform welding (that encompasses all pulse welding processes). So, for different parameters, the average or time-weighted values must be calculated in such cases, for example, for impulse welding, relation (2) can be applied [30,31]:

(2) $$Average\;current=\frac{\left(peak\;current\times peak\;time)+(background\;current\times background\;time\right)}{peak\;time+background\;time}$$

Also, as this approach does not give perfectly accurate results, an alternative way for calculations of heat input is to record and use the “instantaneous power” or “instantaneous energy” of welding arc. The recording of these “instantaneous” values can be performed using a measuring device with high sampling frequency; this device could be a part of the welding power supply or an external piece of independent equipment. In both cases, the sampling frequency must be ten times higher than the waveform frequency [30].

In our research, as described above, the values of voltage and current, representing the average values, were measured. The average current was calculated using relation (2). The value of peak current I_p_ was measured to be 127 A by an ammeter (Table 3, WM 1). The background current I_b_ was of 77 A (60% of peak current). The peak time t_p_ and background time t_b_ were 25%, 50%, and 75% of the welding time. The calculated values of average current exactly coincided with the measured ones and were used as “arc current” in relation (1) to calculate heat input.

**Figure 3:**
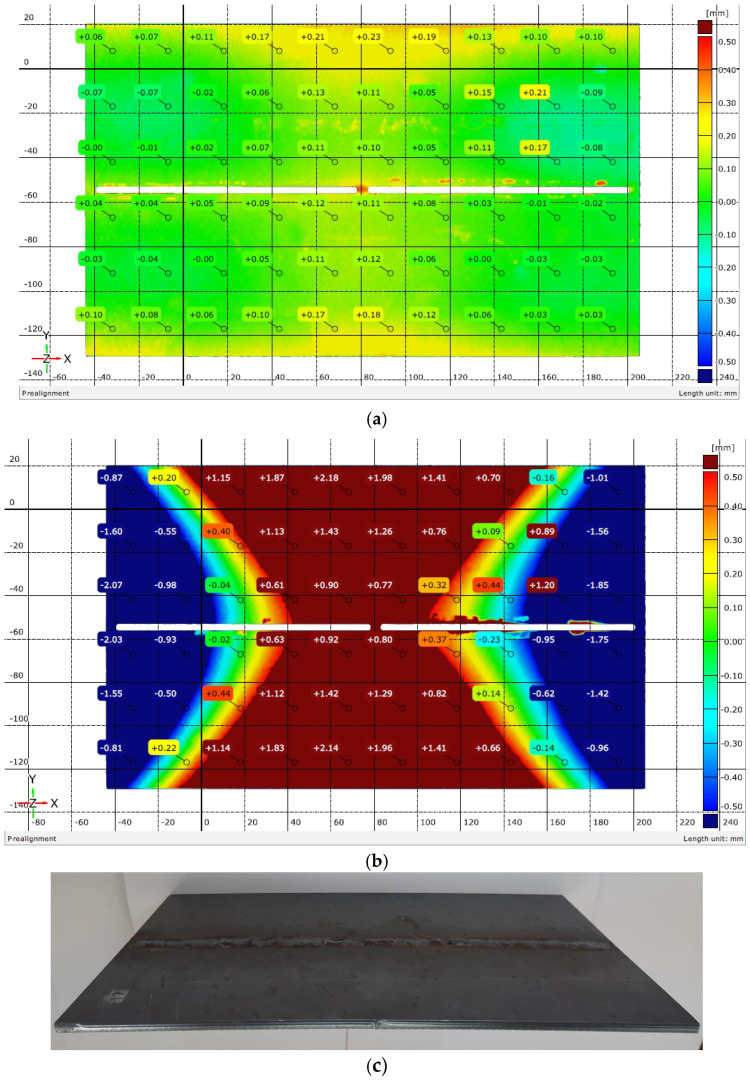
Magnitude of distortions after welding with Fronius: (**a**) welding mode 5; (**b**) welding mode 7; (**c**) appearance of a weldment with upward longitudinal distortion.

**Figure 4:**
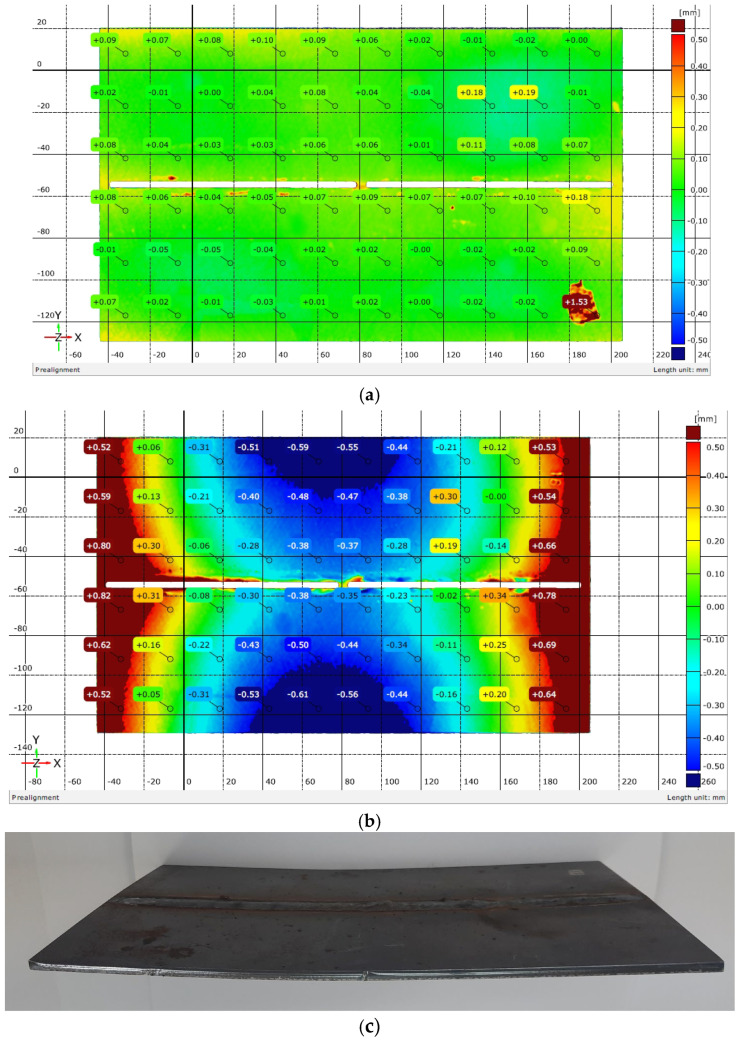
Magnitude of distortions after welding with Astra: (**a**) welding mode 11; (**b**) welding mode 13; (**c**) appearance of a weldment with longitudinal downward distortion.

The distortions of specimens were measured by double-side scanning, prior to and after welding. A 3D laser scanner by Scantech Co., LTD (Hangzhou, Zhejiang Province, China), KSCAN-Magic model, was used. The measurement’s accuracy was defined by the accuracy of the scanner and had a value of 0.01 mm. The software of the scanner gave results as images—color maps. Some of these maps are shown in Figure 3 and Figure 4.

The images from the scanner were superimposed and the changes in dimensions of weldments, compared to raw sheets before welding, were recorded at 60 points. To avoid inaccuracies when superimposing two images, taken before and after welding, the results for the symmetrical points (longitudinal and transverse symmetry with respect to the geometric center of the plates) were averaged.

In this work, only results from points of the periphery of the sheets were presented and analyzed, as the greatest distortions were observed. These peripheral points were located at 12.5 mm from the end of the planks and at 25 mm from each other, as visible in Figure 3 and Figure 4.

## 3. Results

Figure 3 and Figure 4 show the out-of-plane distortions in two of the weldments after cooling. Figure 3c displays an upward longitudinal distortion, while Figure 4c demonstrates a longitudinal downward distortion. According to [11], the observed changes in weldment geometry represent longitudinal bending distortion—the weld line does not coincide with the neutral axis of the weldments, as the longitudinal shrinkage of the weld metal caused bending moments. As transverse distortions in Figure 3a,b and Figure 4a,b were observed, a transverse/angular change also took place during and after welding. The total distortion was a complex one: the biaxial curvature of the weldments led to upwardly curved (Fronius, WM 7) or downwardly curved shapes (Astra, WM 13) like buckling distortion but with only one deformed shape for most of the weldments. According to [25], the distortion, shown in Figure 3c, is a concave–convex type (the neutral plane below the larger part of the weld metal), and in Figure 4c, is a convex–concave type (the neutral plane above the larger part of the weld metal) [25,32].

**Figure 5:**
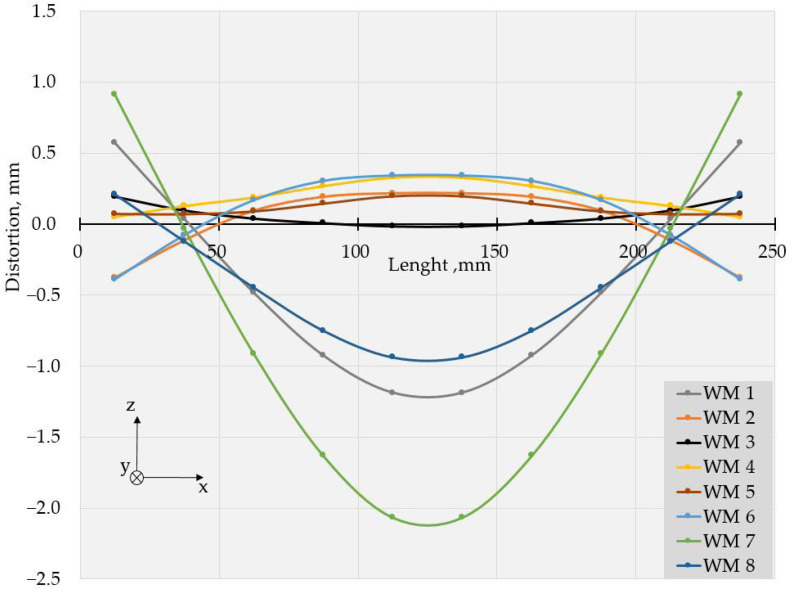
Longitudinal distortions in weldments produced with Fronius using welding modes 1 to 8.

**Figure 6:**
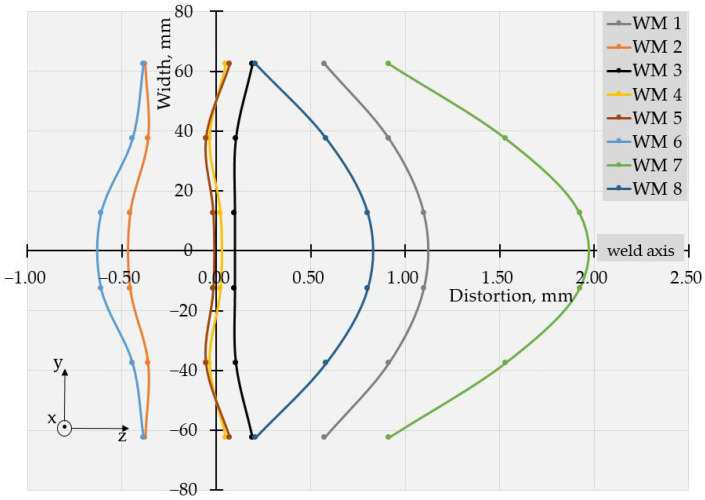
Transverse distortions in weldments produced with Fronius using welding modes 1 to 8.

**Figure 7:**
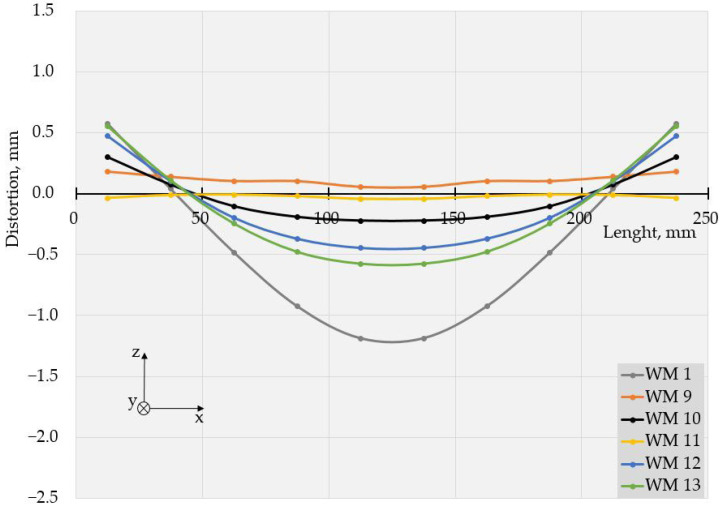
Longitudinal distortions in weldments produced with Astra using welding modes 1, and 9 to 13.

**Figure 8:**
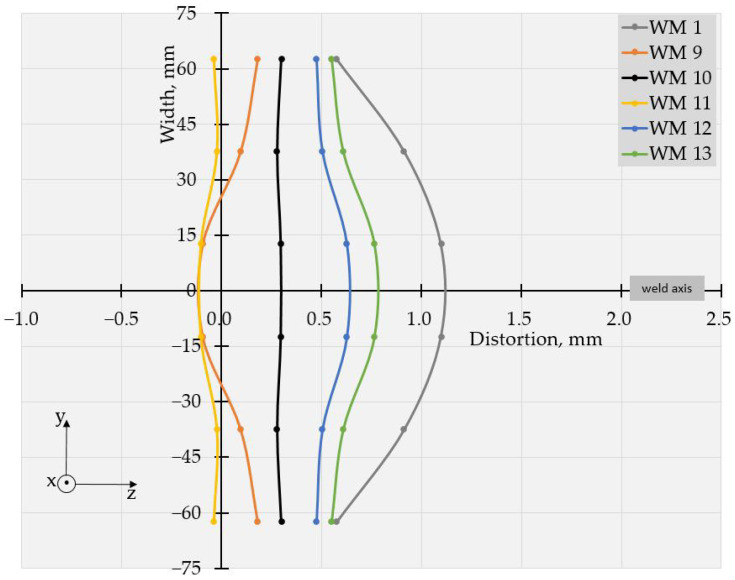
Transversal distortions in weldments produced with Astra using welding modes 1 and 9 to 13.

**Figure 9:**
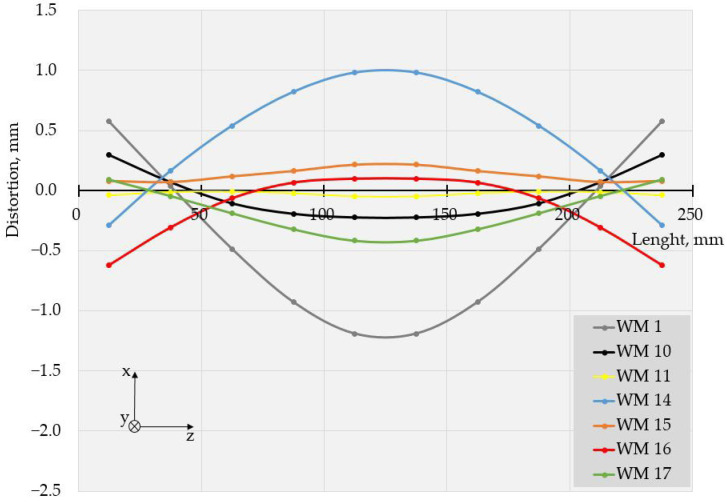
Longitudinal distortions in weldments produced with Astra using welding modes 1, 10, 11, and 14 to 17.

**Figure 10:**
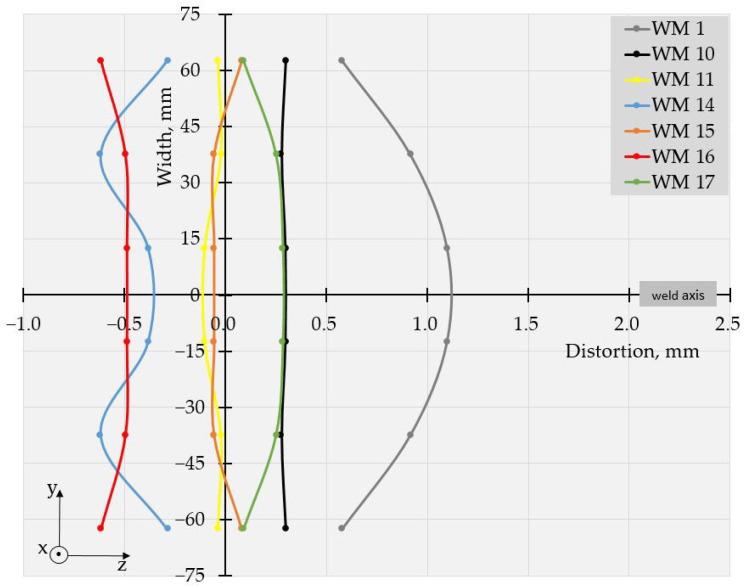
Transversal distortions in weldments produced with Astra using welding modes 1, 10, 11, and 14 to 17.

The values of deflections (displacements in Z direction) [25] are visible in Figure 3 and Figure 4. As the weldments were rectangular, they had two axes of symmetry and one of them coincided with the welding line. Thus, theoretically, if not considering the welding direction, the distortion had to have a symmetrical distribution with respect to those two axes of symmetry. Therefore, to evaluate the welding distortion, the average values of displacements in the Z direction were calculated, using the values of deflection in four points, symmetrical with respect to both axes. This way, 10 values for longitudinal and 6 values for transverse deflection were calculated and were used to draw the graphs, presented in Figure 5, Figure 6, Figure 7, Figure 8, Figure 9 and Figure 10.

Longitudinal distortions in the weldments, made with Fronius, are shown in Figure 5. As these graphs demonstrate, the relationship between the welding distortion and pulse frequency was ambiguous. The same applies to transverse distortion, presented in Figure 6. Nevertheless, a tendency toward greater distortions for weldments made with higher frequencies was observed, and deflections not only increased with pulse frequency but also changed in direction. This way, a transition from the concave–convex mode of distortion (WM2, 5 Hz) to convex–concave mode occurred at a frequency of 20 Hz (WM 3) but the next increase in pulse frequency turned the direction of deflections, i.e., concave–convex distortion appeared again. Another change in the mode of distortion from concave–convex to convex–concave occurred at a frequency of 500 Hz (WM7).

With the increase in frequency, the distribution profile of deflections changed, and for specimens, welded in welding modes 4 and 5 (50 Hz and 100 Hz), in the transverse direction, the distortions resembled buckling distortion [25].

The specimens, welded with Astra, showed similar behavior at the used welding frequencies, as seen in Figure 7, Figure 8, Figure 9 and Figure 10.

The displacements in all specimens, welded in pulse mode, were compared to those in a weldment, made with constant current. It is worth noting that nearly all the pulsed modes led to a reduction in distortion compared to the welding mode with a constant current.

## 4. Discussion

### 4.1. Magnitude of Distortion

The values of the measured deflections, presented in Figure 5, Figure 6, Figure 7, Figure 8, Figure 9 and Figure 10, show that the frequency of the applied current influenced weldment distortion. Distortion in weldments is correlated to residual stresses.

The parameters of pulsed arc welding are peak current I_p_, A, base current I_b_, A, peak time t_p_, s, and base (background) time t_b_, s. The sum of t_p_ and t_b_ equals t—the pulse period, measured in seconds. From these parameters, the following characteristics of a welding mode can be determined [13]: (1) pulse frequency f = I/(t_p_ + t_b_); Hz (2) pulse spacing S = V × t, mm, where V is the welding speed in mm/s; (3) amplitude ratio A = I_b_/I_p_; (4) duration ratio T = t_b_/t_p_; and (5) load duty cycle D = t_p_/t × 100, % [14]. All these characteristics influence residual stresses in weldments and, therefore, influence distortions. In the presented experiments here, only the amplitude ratio was held constant when weldments were made using the power source Astra, and amplitude and load duty cycle were constant when welding was performed with Fronius.

**Figure 11:**
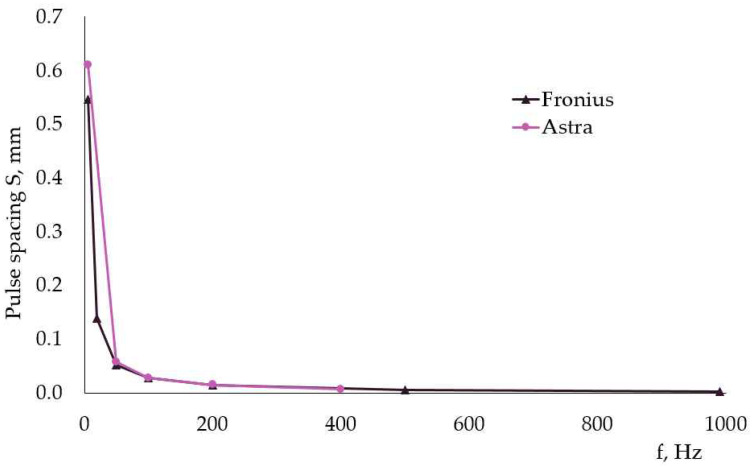
Correlation between current frequency f and pulse spacing S.

To evaluate the influence of the welding parameters on distortions, first pulse spacing S was calculated as a function of welding speed V and pulse period t. The correlation between current frequency f and pulse spacing S is shown in Figure 11 for both power sources. As the frequency increased, the pulse spacing decreased, i.e., these two characteristics should display opposite experimental trends, as described in [13].

**Figure 12:**
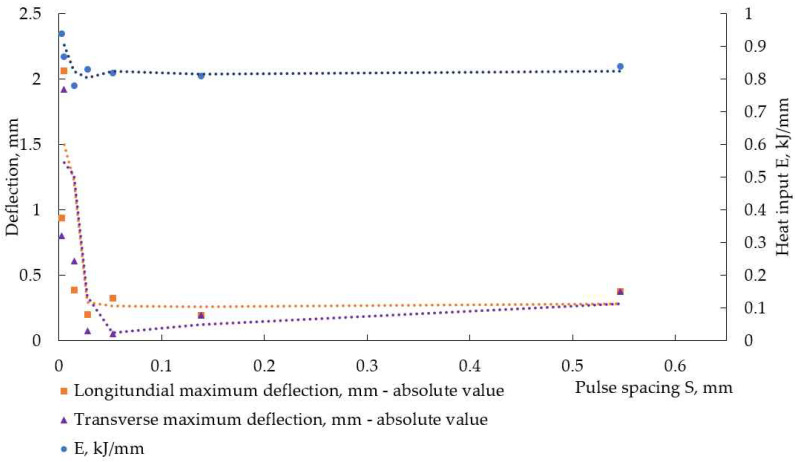
Heat input and absolute maximum values of deflection in the longitudinal direction, measured in weldments, made with Fronius.

**Figure 13:**
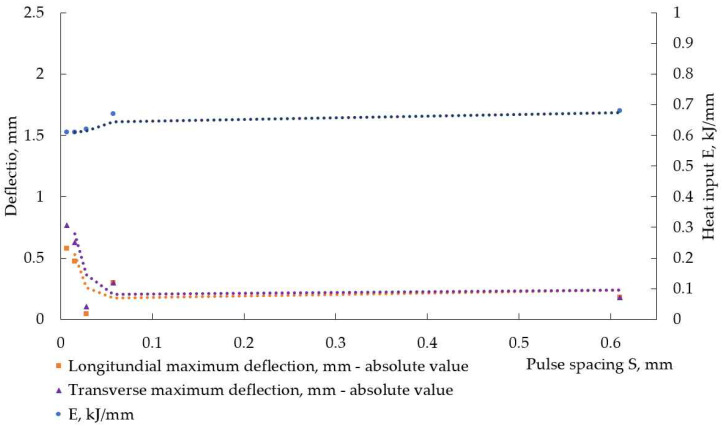
Heat input and absolute maximal values of deflection in longitudinal direction, measured in weldments, made with Astra.

The travel speed (welding speed) was not constant for different welding modes, and this complicated the analysis of the experimental results, as heat input per unit length was another variable. This is visible in Table 4 and Figure 12 and Figure 13. These figures represent the change in heat input E and maximum distortion with pulse spacing S. In weldments, made with Fronius, the energy, transferred to weldments, was higher at smaller pulse spacing (higher frequency).

During pulse welding, a double fusion of weld occurs. In pulsed arc welding, the consumable electrode melts within the peak current time by the arc heat to form droplets. Then, the droplets detach and transfer to the welded parts and mix with them, forming the weld pool. The droplet transfer mainly occurs during peak time [12,16]. During the base time, the temperature drops, partial solidification of the weld takes place, and the next peak current leads to its melting again. A lower pulse spacing means higher frequency, i.e., less time for the melted metal to solidify. It is reported in [13] that a greater pulse spacing during pulsed gas tungsten arc welding can decrease the degree of double fusion of welds and that the extent of double fusion is related to the heat input. The authors of [13] have found that, during pulsed gas tungsten arc welding, a greater pulse spacing leads to lesser heat input and reduces residual stresses in weldments of stainless steel. A similar trend for the relationship between heat input and pulse spacing was observed in our experiment. The trend toward higher heat input was very pronounced at smaller pulse spacings in weldments, produced with Fronius, and this is visible in Figure 12 and in Table 4: the heat input reached a minimum at pulse spacing of 0.0147 mm at f = 200 Hz, where the welding speed was the highest (the lowest welding time). The next increase in heat input with pulse spacing can be attributed to the lower welding speeds, and this is a manifestation of the inability of manual arc welding to ensure accurate control of welding parameters compared to mechanized welding. The data in Table 4 suggest that, for both power sources, welding speed was an important factor for heat input. But welding speed is already included in the pulse spacing equation—increasing welding speed at constant frequency should increase pulse spacing and decrease heat input. The pulse period, that is, the inverse of frequency, is the second factor in the pulse spacing equation—increasing frequency at constant welding speed should decrease pulse spacing and increase the heat input. Thus, two antagonistic factors that influence the heat input in opposite directions with their simultaneous increase can be described: (1) welding speed and (2) current frequency. It is obvious from the data, collected during our experiments, that the welding speed, i.e., the time the weldments were under the influence of the arc, had prevalent action on heat, transferred to the weldments, at a constant load duty ratio of 50%. This is a conclusion drawn from the data in Figure 12 and Figure 13 and Table 4.

In [33], it is reported that an increase in pulse spacing increases the angular distortion in weldments of stainless steel welded by pulsed gas tungsten arc welding. At smaller pulse spacing, i.e., higher current frequencies, a narrower heat-affected zone is formed, and the ratio between weld depth and width increases. This leads to a smaller temperature gradient between the weld metal and base metal, thus reducing thermal strains and stresses, and induced from them welding distortions. In the experiment presented here, the welding speed was not constant and influenced heat input as well as heat distribution. At lower welding speeds, the weldments were subjected to the thermal influence of the welding arc for longer, and despite the higher current frequencies, wider welds had to form and, therefore, the higher temperature difference between the weld metal and base metal along the welded plates arose, leading to increased distortion. This is observed in Figure 12 and Figure 13. Thus, the travel speed of the welding electrode proved its importance to the welding process once again.

**Figure 14:**
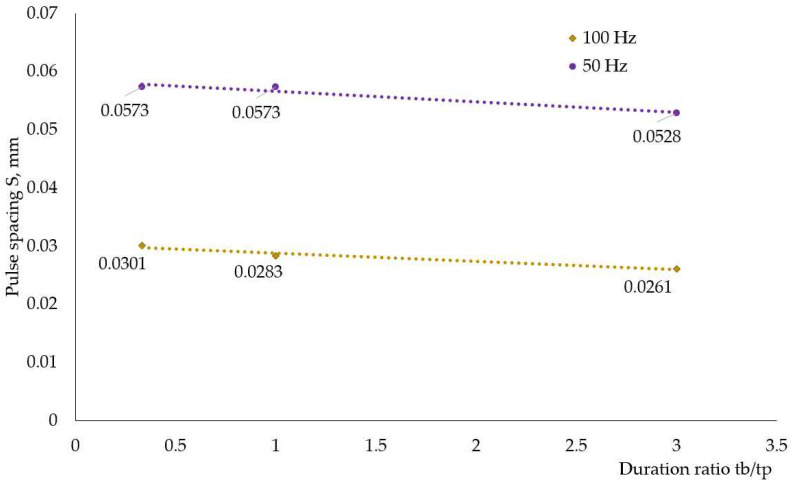
Pulse spacing at different duration ratios for weldments produced with Astra.

**Figure 15:**
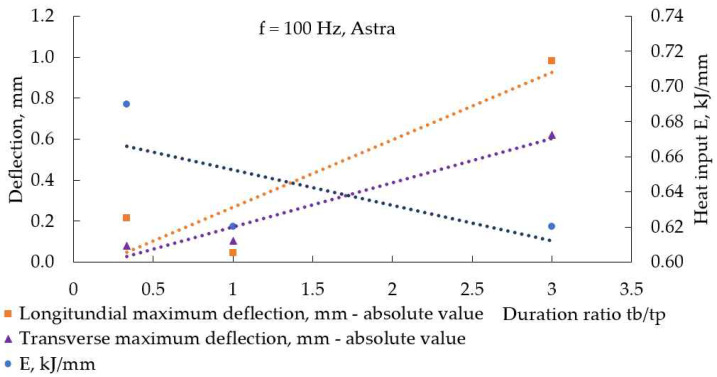
Heat input and absolute maximal values of deflection in the longitudinal direction, measured in weldments, made with Astra at 100 Hz and different duration ratios (load duty cycle).

**Figure 16:**
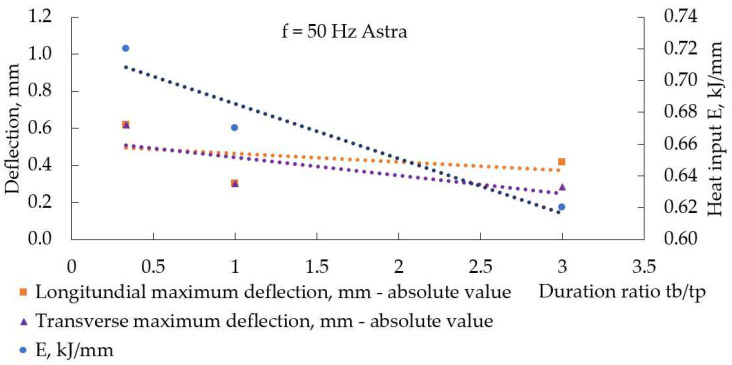
Heat input and absolute maximal values of deflection, measured in weldments, made with Astra at 50 Hz and different duration ratios (load duty cycle).

Figure 14 demonstrates how the duration ratio (inversely proportional to the load duty cycle) affected pulse spacing at a constant frequency. The shorter the base current acted, the greater the pulse spacing was; in other words, the welding speed was higher. Counterintuitive to this, the highest heat input for welding modes at 100 Hz and 50 Hz was calculated for the smallest duration ratio, as Figure 15 and Figure 16 demonstrate, and this fact reflects the longer time the welding arc had at peak current to transfer heat to the weldments at smaller duration ratios.

It is described in [34] that, at the weld region, longitudinal residual welding stresses are tensile and close to the yield point, but away from the weld, the base metal is characterized by residual compressive stresses. A reduction in heat input results in a narrower area near the weld, where tensile stresses act, and a reduction in compressive stresses on the base metal. At the same time, reducing heat input can result in a higher temperature gradient, and this way, a reduction in heat input causes increased distortions [33]. This bidirectional influence of heat input on welding distortions is demonstrated in Figure 15 and Figure 16.

### 4.2. Mode of Distortion

The works [25,32] describe that the mode of distortion depends on heat input and its influence on temperature gradient through the plate thickness. When heat input is relatively small, the differences in peak temperature between the top and the bottom of a weldment are relatively large, angular distortion is significant, and concave–convex distortion is observed after welding [25]. In our experiments, the concave–convex mode of distortion was observed for pulse spacings, corresponding to relatively small heat input and high frequencies, and this fact confirms the findings of [25,32]. With the increase in heat input, the temperature gradient across the welded plate decreased, angular distortions were small, and convex–concave distortions were observed after welding. The changes in distortion mode can be seen in Figure 5, Figure 6, Figure 7, Figure 8, Figure 9 and Figure 10.

## 5. Conclusions

When manual metal arc welding is used, the resulting weldment is a product of a system “welding power source—welding parameters—welder’s qualification and performance—welded material”. Understanding the interconnectedness of a welding system is crucial for gaining a holistic view of how a weldment is affected by changes in this system. Among the parts of a manual welding system, the most unstable is the welder’s qualification and performance, as the welder can always change, and the welder’s hand does not possess the immutability of a robot hand. This fact complicated the analysis of the experimental results presented here. Thus, the principal conclusion that can be drawn is the necessity for a prior adjustment of welding parameters for minimum welding distortions.

The work in the pulsed mode during manual arc welding affects the arc stability and welding speed and, therefore, the energy input. Increasing current frequency during manual metal arc welding does not impact heat input and is connected to welding distortions in a straightforward manner.

Despite the limitations in welding speed control during manual arc welding, the results presented here allow a tendency toward greater distortions at higher (above 200 Hz) current frequencies to be outlined.

The load duty cycle is another welding parameter that influences welding speed and heat input. Its action on welding distortions during pulsed manual arc welding is complicated and not unidirectional.

The literature data on pulsed manual arc welding is limited and the few publications on this topic suggest that current frequencies higher than 10 Hz do not allow accurate control of the weld pool. However, the experimental results in this work demonstrate that current frequencies as high as 200 Hz can decrease welding distortions to an even lesser extent than low welding current frequencies.

## Figures and Tables

**Table 1:** Chemical composition of used steel.

Chemical Composition of S235JR in Weight %								
	C	Si	Mn	S	P	N	Cu	Other
S235JR EN 10025-2:2019	max 0.17	-	max 1.40	max 0.035	max 0.035	max 0.012	max 0.55	-
Quant metric analysis	0.11	0.03	0.43	0.012	0.017	-	0.022	-

**Table 2:** Main characteristics of the used power sources.

	Trademark	FRONIUS		REDKO	
Parameters					
Model		TransPocket 180		Astra 200 AC/DC PULSE LSD	
Input power		1 × 230 V, 50/60 Hz		1 × 220/230/240 ± 10% V, 50/60 Hz	
		MMA	TIG	MMA	TIG
Rated input current (A)		25 AC/DC	25 AC/DC	28/32AC/DC	19/21 AC/DC
Rated input power (KW)		5.75 AC/DC	5.75 AC/DC	6.4/7.2AC/DC)	4.3/4.8 AC/DC
Welding current for welding (40 °C, 10 min)		40% 180 A	40% 220 A	40% 200 A	40% 200 A
		60% 150 A	60% 150 A	60% 140 A	60% 140 A
		100% 120 A	100% 120 A	100% 110 A	100% 110 A
Pulse Frequence (HZ)		0.5~990	0.5~990	0.5~400	0.5~999
No-load voltage (V)		101		67	
Net Weight (kg)		8.7		17	

**Table 3:** Parameters of the used welding modes.

Power Source	Welding Mode (WM) No.	Set Current, I, A	Measured Effective Current I, A	Voltage/Average Value U, V	Frequency of Pulses f, Hz	Welding Time t, s	Linear Energy E, kJ/mm
Average TransPocket 180 and ASTRA 200	1	120	127	28…30/29	-	71	0.84
TransPocket 180 Duty Cycle = 50%	2	120	102	28…32/30	5	86	0.84
	3	120	102	28…30/29	20	85	0.81
	4	120	102	27…29/28	50	90	0.82
	5	120	102	29…31/30	100	85	0.83
	6	120	102	28…32/30	200	80	0.78
	7	120	102	26…30/28	500	95	0.87
	8	120	102	29…31/30	990	96	0.94
ASTRA 200 AC/DC PULSE LCD	9	120	102	26…28/27	5	77	0.68
	10	120	102	24…26/25	50 Duty Cycle–50%	82	0.67
	11	120	102	22…24/23	100 Duty Cycle–50%	83	0.62
	12	120	102	23…25/24	200	78	0.61
	13	120	102	21…23/22	400	85	0.61
	14	120	90	22…26/24	100 Duty Cycle–25%	90	0.62
	15	120	115	22…26/24	100 Duty Cycle–75%	78	0.69
	16	120	115	23…25/24	50 Duty Cycle–75%	82	0.72
	17	120	90	22…26/24	50 Duty Cycle–25%	89	0.62

**Table 4:** Some characteristics of pulsed welding modes and maximum values of welding distortions.

	**Welding Mode**	**f, Hz**	**V, mm/s**	**S, mm**	**E, kJ/mm**	**Longitudinal Maximum Deflection, mm—Absolute Value**	**Transverse Maximum Deflection, mm—Absolute Value**
Fronius, D = 50%	8	990	2.45	0.0025	0.94	0.9375	0.8025
	7	500	2.47	0.0049	0.87	2.065	1.925
	6	200	2.94	0.0147	0.78	0.3875	0.61
	5	100	2.76	0.0276	0.83	0.1975	0.0725
	4	50	2.61	0.0522	0.82	0.3275	0.05
	3	20	2.76	0.1382	0.81	0.1925	0.1925
	2	5	2.73	0.5465	0.84	0.3775	0.3775
Astra, D = 50%	13	400	2.76	0.0069	0.61	0.5775	0.765
	12	200	3.01	0.0151	0.61	0.475	0.6275
	11	100	2.83	0.0283	0.62	0.045	0.1025
	10	50	2.87	0.0573	0.67	0.3	0.3
	9	5	3.05	0.6104	0.68	0.18	0.18
	**Welding Mode**	**Duration Ratio (Load Duty Cycle D)**	**V, mm/s**	**S, mm**	**E, kJ/mm**	**Longitudinal Maximum Deflection, mm—Absolute Value**	**Transverse Maximum Deflection, mm—Absolute Value**
Astra, f = 100 Hz	14	3 (25%)	2.61	0.0261	0.62	0.9825	0.62
	11	1 (50%)	2.83	0.0283	0.62	0.045	0.1025
	15	0.3 (75%)	3.01	0.0301	0.69	0.215	0.08
Astra, f = 50 Hz	17	3 (25%)	2.64	0.0528	0.62	0.415	0.285
	10	1 (50%)	2.87	0.0573	0.67	0.3	0.3
	16	0.3 (75%)	2.87	0.0573	0.72	0.6175	0.6175

## Data Availability

Data are contained within the article.
